# Use of viscoelastic substance for preventing Descemet’s membrane rupture in deep anterior lamellar keratoplasty

**DOI:** 10.5935/0004-2749.20210037

**Published:** 2021

**Authors:** Yusuf Koçluk, Burcu Kasım, Emine Alyamaç Sukgen

**Affiliations:** 1 Department of Ophthalmology, Adana City Training and Research Hospital, Adana, Turkey

**Keywords:** Descemet’s membrane/surgery, Viscoelastic substance, Corneal transplantation, Corneal stroma, Keratoplasty, penetrating, Lâmina limitante posterior/cirurgia, Substâncias viscoelásticas, Transplante de córnea, Substância propria, Ceratoplastia penetrante

## Abstract

**Purpose:**

This study aimed to investigate the effect of using a viscoelastic substance in Descemet’s membrane rupture in “double bubble” deep anterior lamellar keratoplasty.

**Methods:**

The medical records and videos of surgeries of 40 patients who underwent surgery between January 2014 and July 2015 were retrospectively evaluated. The patients were divided into two groups: 20 patients whose perforation of the posterior stromal wall was performed without administration of any viscoelastic substance (group 1) and 20 patients whose perforation of the posterior stromal wall was performed with administration of viscoelastic substance onto the posterior stroma (group 2). The Descemet’s membrane perforation rate was compared between groups.

**Results:**

Perforation of the Descemet’s membrane was observed in 12 (60.0%) patients in group 1 and only three (15.0%) patients in group 2. This difference was statistically significant (p=0.003). Only one (5%) patient in group 2 had macroperforation during the procedure, and the surgery was converted to penetrating keratoplasty. Eleven (55.0%) patients in group 1 had macroperforation of Descemet’s membrane, and surgeries were converted to penetrating keratoplasty. This difference between the groups was statistically significant (p=0.001).

**Conclusions:**

Administering a viscoelastic substance onto the posterior stromal side just before puncture is an effective method to decrease the risk of Descemet’s membrane perforation in deep anterior lamellar keratoplasty.

## Abstract (pt)

**Objetivo:**

Investigar o efeito do uso de uma substância viscoelástica na ruptura da membrana de Descemet em casos de ceratoplastia lamelar anterior profunda em “bolha dupla”.

**Métodos:**

Foram avaliados retrospectivamente prontuários e vídeos de cirurgias de 40 pacientes operados entre janeiro de 2014 e julho de 2015. Os pacientes foram divididos em dois grupos: 20 pacientes nos quais a parede posterior do estroma foi puncionada sem a colocação de nenhuma substância viscoelástica (grupo 1) e 20 pacientes nos quais uma substância viscoelástica foi aplicada sobre o estroma posterior ao ser puncionada a parede posterior do estroma (grupo 2). A taxa de perfuração da membrana de Descemet foi comparada entre os grupos.

**Resultados:**

Observou-se perfuração da membrana de Descemet em 12 casos (60,0%) no grupo 1 e em apenas 3 casos (15,0%) no grupo 2. Essa diferença foi estatisticamente significativa (p=0,003). Apenas um caso (5%) no grupo 2 teve macroperfuração durante o procedimento, sendo a cirurgia então convertida em uma ceratoplastia penetrante. Onze casos (55,0%) no grupo 1 tiveram macroperfuração da membrana de Descemet e essas cirurgias foram convertidas em ceratoplastias penetrantes. Essa diferença entre os grupos foi estatisticamente significativa (p=0,001).

**Conclusões:**

A aplicação de substância viscoelástica sobre o lado posterior do estroma logo antes da punção é um método eficaz para diminuir o risco de perfuração da membrana de Descemet na ceratoplastia lamelar anterior profunda.

## INTRODUCTION

Treatment of many corneal stromal pathologies, such as keratoconus, corneal scars, stromal dystrophies, and degenerations, is currently performed with deep anterior lamellar keratoplasty (DALK) as a surgical option^(1-5)^. DALK has gradually become a popular alternative to penetrating keratoplasty (PKP) for patients who have corneal diseases with healthy Descemet’s membrane (DM) and endothelium.

There are various descriptions of DALK techniques. In the “big bubble” technique, forceful air injection is performed into the deep stroma to obtain cleavage separation of the DM from the overlying stroma, with formation of a large air bubble between these two layers^(6,7)^. In another technique, “double bubble” DALK, the formation of the large bubble can be identified by the small bubbles in the anterior chamber, and this technique potentially increases the success of the completion of the procedure as DALK, especially in patients with stromal opacities^(8)^.

In DALK, the ratio of intraoperative complications may vary depending on the surgeon and applied surgical technique. According to a study, perforation of DM is the most common intraoperative complication of DALK in the early phase of the learning curve^(9)^. In the same study, postoperative complications included double anterior chamber in cases with microperforation of DM. Moreover, the surgery has been converted to PKP in cases with macroperforation. Thus, it is necessary to decrease the perforation of DM in DALK using new techniques.

In the present study, the effect of a viscoelastic substance (VES) on DM rupture in “double bubble” DALK was investigated in the consecutive surgeries.

## METHODS

This retrospective clinical study was conducted in an ophthalmology clinic of a tertiary care center after obtaining approval from the hospital ethics committee (ANEAH, EK.2016/82) and written informed consent from all patients. The first 40 consecutive patients who underwent “double bubble” DALK in which big bubble formation could be obtained in cases of keratoconus and corneal stromal dystrophy were included in this study. Corneal buttons were obtained from the hospital’s eye bank.

The medical records and videos of surgeries of the 40 patients who underwent surgery on between January 2014 and July 2015 were evaluated. The patients were divided into two groups: first 20 consecutive patients whose perforation of the posterior stromal wall was performed without administration of any VES (group 1) and subsequent 20 patients whose perforation of the posterior stromal wall was performed with administration of VES onto the posterior stroma (group 2). Patients with healed corneal hydrops and DM scars were excluded from the study. Moreover, patients in which big bubble formation could not be obtained by air and DALK could be completed by manual technique were excluded.

### Surgical technique

“Double bubble” DALK was performed in group 1, as previously described^(8)^. As distinct from that technique, VES was administered on the posterior stroma before the posterior stroma was perforated in group 2. Moreover, 1.4% sodium hyaluronate (Bio-Hyalur Plus, Bio-tech Vision Care Pvt. Ltd., Gujarat, India) was used as VES. All surgeries were performed under general anesthesia by the same surgeon.

**Figure 1:**
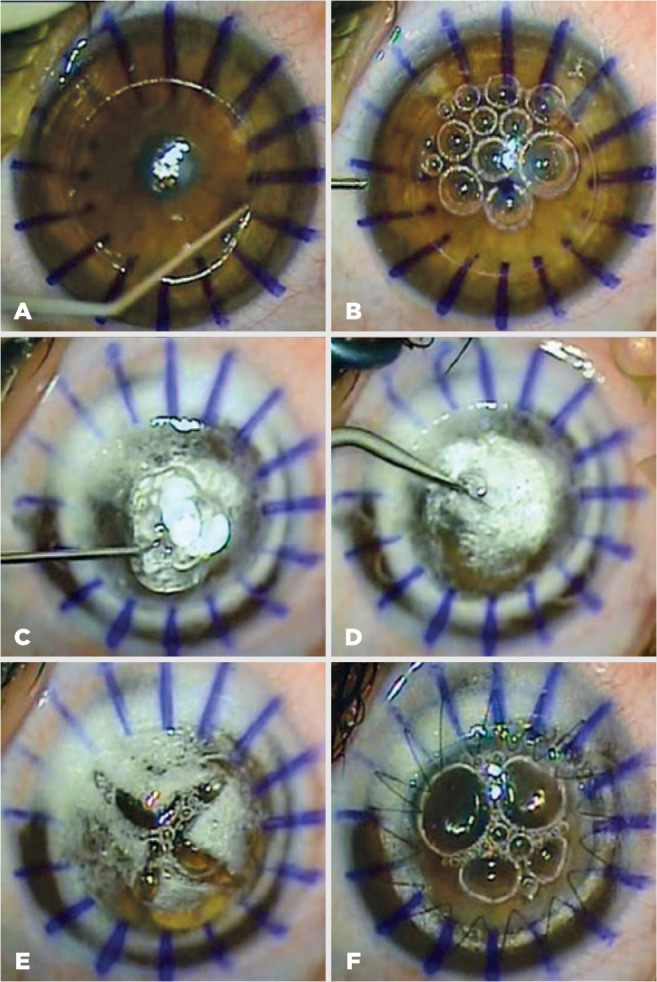
Images from the surgical steps of DALK. (A) Partial-thickness trephination of the recipient cornea, (B) a small amount of air of 3-4 mm in diameter was injected into the AC, (C) VES was placed onto the roof of the air bubble, (D) posterior stroma was punctured by carefully using a 20-G MVR knife (Alcon, USA) in group 2, (E) the air bubble initially injected into the AC was maintained in the AC, (F) 10-0 monofilament nylon was used to suture the donor lenticule.

A vacuum trephine (Katena Products, Inc. Denville, New Jersey, USA) was used to perform partial-thickness trephination of the recipient cornea to an approximate depth of 60-70% of the corneal thickness based on the measurements obtained by Pentacam corneal topography (Figure 1A). Then, paracentesis was performed posterior to the limbus at 11 o’clock, and the aqueous was allowed to escape to lower the intraocular pressure (IOP). From this paracentesis, air of 2-3 mm in diameter was injected into the anterior chamber (AC) (Figure 1B).

DALK= deep anterior lamellar keratoplasty; AC= anterior chamber; VES= viscoelastic substance.

The air was injected into the corneal stroma using a 27-G disposable needle attached to a 5-mm syringe, containing sterile air. The needle was bent at an angle, approximately 80°, close to its base in a track away from the bevel. In a bevel-down position, the needle tip was progressed tangentially into the paracentral corneal stromal tissue at a depth of 70-80% through the partial trephination wound. Firm and consistent pressure was used as the air was injected via the syringe. Initially, intrastromal blanching was observed; then, the separation wave of the DM from the stroma was noted. Finally, formation of air bubble was observed and confirmed by the displacement of the previously injected small AC bubble to the periphery.

A disposable crescent knife was used to dissect and remove the anterior part of the corneal stroma to expose the posterior portion, overlying the big air bubble. A point on the central posterior stromal surface was stained with gentian violet. This stained point, which was the roof of the air bubble, was carefully punctured using a 20-G microvitreoretinal blade (MVR: Alcon Laboratories, Inc., Fort Worth, TX, USA) in group 1. In group 2, VES, approximately 3×3×3 mm in size, was placed on this stained point just before perforation of the posterior stroma (Figure 1C). Then, stromal puncture was performed through this VES (Figure 1D). The VES was injected through this opening, into the space between the posterior stroma and DM. The thin layer of posterior corneal stromal tissue was divided into four quadrants using a pair of curved blunt-tipped scissors. Baring the DM completely, each quadrant was subsequently ex cised. During the surgery, the initially injected air bubble was maintained in the AC (Figure 1E).

DM of the 0.25-mm oversized donor cornea was removed after staining with 0.06% trypan blue. All VES was washed away with balanced salt solution from the DM of the host cornea before suturing the donor graft with 10-0 monofilament nylon (Figure 1F).

The surgeries where macroperforation of DM developed were converted to PKP.

### Statistical analysis

Data analysis was performed using the Statistical Package for Social Sciences for Windows software (SPSS version 16.0, SPSS Inc. Chicago, USA). The normality distribution of the variables was tested using the Kolmogorov-Smirnov test. The descriptive statistics of normally distributed continuous variables (age, IOP, visual acuity, donor age, and graft and recipient sizes) were expressed as mean ± standard deviation, and descriptive statistics of abnormally distributed variables were expressed as median (minimum-maximum). Between the groups, normally distributed variables were compared using Student’s t-test, and abnormally distributed variables were compared using Mann-Whitney U test. Categorical variables were presented as frequency (%) and compared between the groups using chi-square test and Fisher’s exact test. Differences were considered statistically significant when the p value was <0.05.

## RESULTS

The present study included 40 eyes of 40 patients who underwent “double bubble” DALK. The mean age of the patients during surgery was 36.9 ± 1.07 years in group 1 and 38.2 ± 1.11 years in group 2 (p=0.709). The preoperative IOP was not statistically significantly different in the two groups (p=0.951) and was in normal range in all patients. In both groups, all patients were phakic. The preoperative findings are summarized in table 1.

**Table 1:** Preoperative findings

	Group 1	Group 2	P-value
Age (years)	36.9 ± 1.07	38.2 ± 1.11	0.709
Sex (Female/male)	10/10	11/9	0.752
BCVA	0.03 ± 0.01	0.03 ± 0.02	0.434
Recipient size (mm)	7.38 ± 0.23	7.40 ± 0.24	0.871
Graft size (mm)	7.63 ± 0.23	7.65 ± 0.24	0.871

BCVA= best corrected visual acuity (measured using a Snellen chart, recorded in decimal notation).

The preoperative diagnosis of corneal pathology was not statistically significantly different in the two groups (p=0.796). In group 1, eight (40.0%) patients had keratoconus, eight (40.0%) had macular corneal dystrophy, and four (20.0%) had lattice corneal dystrophy. In group 2, six (30.0%) patients had keratoconus, nine (45.0%) had macular corneal dystrophy, and five (25.0%) had lattice corneal dystrophy.

Perforation of DM was observed in 12 (60.0%) patients in group 1 and three (15.0%) patients in group 2. This difference was statistically significant (p=0.003). The stages where DM perforation occurred in both groups during DALK are presented in table 2. In group 1, DM perforation was observed during posterior stromal wall puncture in 9 (75.0%) of 12 patients who had DM rupture. In contrast, there was no perforation of the DM at this stage of surgery in group 2. Only one (5%) patient had macroperforation during the procedure in group 2, and the surgery was converted to PKP. Eleven (55.0%) patients in group 1 had macroperforation of DM, and these surgeries were also converted to PKP. This difference between the groups was statistically significant (p=0.001).

**Table 2:** Distribution of DM perforation in groups

	Group 1	Group 2	P-value^*^
Presence of DM perforation	12/20 (60%)	3/20 (15.0%)	0.003
Phase of DM perforation			0.036
In the course of posterior stromal wall puncture	9/20 (45.0%)	0/20 (0%)	0.001
During removal of the posterior stromal pieces	2/20 (10%)	1/20 (5%)	0.500
During graft suturing	1/20 (5%)	2/20 (10%)	0.500
Number of patients who had macroperforation of DM and conversion to PKP	11/20 (55.0%)	1/20 (5.0%)	0.001

* Chi-square test.

DM= Descemet’s membrane; PKP= penetrating keratoplasty.

## DISCUSSION

Despite some intraoperative complications, DALK is the logical alternative for the surgical treatment of keratoconus and corneal stromal opacification with a functional endothelium. While endothelial rejection is the most common cause of graft rejection, which may lead to graft failure, DALK reduces that risk by protecting the host endothelium. However, DALK is a longer and technically more demanding procedure. Therefore, the main drawback is its long learning curve^(10-13)^.

The choice of the surgical technique and the surgeon’s learning curve play probably the most important role in different rates of perforation of DM and conversion to PKP^(14)^. Previous studies have shown the timing of perforation of DM in different stages, such as initial trephination, during initial air injection by the needle itself and during dissection of the posterior stroma^(14)^. In a previous study, the major cause of perforation of DM was injection of excess air, which was observed in 50% of cases^(15)^.

To the best of our knowledge, no other study investigated the prevention of perforation of DM in DALK. However, several studies reported the complications of DALK^(9,14,15)^. These studies on perforation of DM were different from the current study. In the current study, perforation of DM was observed most commonly in the course of posterior stromal wall puncture. Thus, VES was used to prevent perforation of DM at that stage of surgery.

DALK has more advantages than PKP, especially at the postoperative follow-up. Therefore, it is important to complete the surgery as DALK with an intact DM. The current study reported a useful and effortless technique in DALK to prevent DM rupture, especially for beginners in DALK.

The effect of the VES on DM perforation was evaluated in this study. Perforation of the DM seemed to be the most common and most severe complication reported in the literature, with an incidence rate varying from 9% to 28%^(14-18)^. It can develop during the different steps of the DALK procedure. We can divide these steps into three sections. The first is in the course of posterior stromal wall puncture, the second is during the removal of the posterior stromal pieces, and the third is during graft suturing. However, there are few studies on the phase when DM perforation can develop and affecting factors. This study investigated the proportion and distribution of DM perforations in 40 patients who underwent “double bubble” DALK.

In 20 patients (Group 1), in the course of posterior stromal wall puncture phase, puncture was performed without administration of VES in the stromal side. Perforation was observed in 12 patients (60%) in this group, and 9 (75.0%) of them developed perforation during stromal puncture with MVR knife. In this phase, the DM was perforated by MVR knife due to anterior movement of the DM with the sudden release of the big bubble between the DM and posterior stroma.

The proportion of DM perforation in group 1 was higher than those in other studies, and the surgical procedure was changed only at the stage of posterior stromal wall puncture. Just before performing stromal puncture, a VES was administered onto the central of posterior stromal surface in the subsequent 20 patients (group 2); then, puncture was performed. None of the patients in this group developed DM perforation at this stage. The tamponade effect of the VES disabled the sudden release of the big bubble and the sudden anterior movement of the DM. As a result, no DM rupture occurred at this stage.

Conversion to PKP is mandatory if macroperforation of DM has occurred. Small and/or peripheral perforations can be ignored, and the procedure can be continued in normal fashion. Central microperforations should be evaluated individually to assess whether it is safe to continue with DALK or convert to PKP^(9,19,20)^. In group 1, DM perforation was noted at the central part of DM, and conversion to PKP was preferred in these nine patients. In one (5%) patient in group 1 and two (10%) patients in group 2, microperforation was observed during graft suturing. DALK was continued and completed with the help of air injection into the AC in these patients.

Group 1 consisted of previous patients of the same surgeon who is in the learning curve, which could be considered as one of the drawbacks of this study. In addition, previous studies have reported that the air bubble roof is punctured at the center of the cornea using a 15° knife^(7-9,21)^. An MVR blade was used to puncture the roof of the air bubble for all patients. In group 1, the higher proportion of DM perforation than was indicated in the related studies could be the result of the use of an MVR blade because both sides of the MVR blade are sharp, thus increasing the DM perforation risk at this stage. However, the VES used in group 2 helped prevent this increased risk.

Intraoperative optic coherence tomography may also be a useful tool in making the surgical decisions in various steps of DALK and increasing the safety of surgery^(22)^. However, its use mainly facilitates the creation of the big bubble by assessing the depth of trephination and needle insertion^(23)^.

Therefore, the application of the VES on the posterior stromal side just before the puncture is an effective method for decreasing DM perforation risk during posterior stroma puncture in DALK.
